# 537 Burned at Sea: Transport and Critical Care of Two Sailors Injured on the Pacific Ocean

**DOI:** 10.1093/jbcr/irae036.171

**Published:** 2024-04-17

**Authors:** Jill M Cancio, Matthew Tadlock, Leopoldo C Cancio

**Affiliations:** US Army Institute of Surgical Research, Floresville, TX; Naval Medical Center San Diego, San Deigo, CA; US Army Institute of Surgical Research, Fort Sam Houston, TX; US Army Institute of Surgical Research, Floresville, TX; Naval Medical Center San Diego, San Deigo, CA; US Army Institute of Surgical Research, Fort Sam Houston, TX; US Army Institute of Surgical Research, Floresville, TX; Naval Medical Center San Diego, San Deigo, CA; US Army Institute of Surgical Research, Fort Sam Houston, TX

## Abstract

**Introduction:**

Burns have constituted 5-10% of combat casualties from recent conflicts and are projected to become even more common during future war at sea. Advances in critical care and surgery have improved postburn mortality. Nevertheless, survivors of such massive injuries require immense efforts by an interprofessional team from point of injury through the restoration of long-term function and quality of life. Here, we documented the challenges and resources required for 2 sailors who were severely burned at sea on the Pacific Ocean and who received definitive multidisciplinary care at an American Burn Association-verified Burn Center.

**Methods:**

In July 2022, 2 sailors from a coalition nation sustained life-threatening burns during a boiler-room fire aboard a ship at sea during the RIMPAC multinational exercise on the Pacific Ocean. Multiple naval assets including the US Coast Guard, the French Navy, and the US Navy collaborated to rescue and evacuate these patients. A Burn Flight Team transported the casualties aboard US Air Force aircraft to an American Burn Association-verified Burn Center in the United States. Both sailors returned to their home country approximately 5.5 months postburn. Unless otherwise stated, data are presented as means.

**Results:**

Patient 1 was 27 years old with an 86% TBSA burn; patient 2 was 29 years old with a 70% burn. Both sustained concomitant inhalation injury. Transfer from point of injury to definitive care took 11 days with 7 handoffs. Mean hospital length of stay (LOS) was 101 days, ICU LOS 50 days, and ventilator days 10.5. They underwent an average of 14 major surgical operations and were transfused 78 units of blood products. They received an average of 4.4 hours of direct rehabilitation per day during inpatient hospitalization, and as outpatients, they received an average of 63 rehabilitation treatment sessions.

**Conclusions:**

The care of these 2 severely burned sailors required the coordination of naval assets from multiple countries and lengthy resource-intense definitive care at the Burn Center. These efforts took place during peacetime and were not complicated by a combat environment to include communication and air transport challenges.

**Applicability of Research to Practice:**

Future peer and near-peer combat operations, to include war at sea, are expected to generate greater numbers of burn casualties than were seen during recent conflicts. Properly forecasting and preparing for the substantial needs of those future casualties is an essential component of readiness.

